# Congenital hydrocephalus in an Egyptian baby with trisomy 18: a case report

**DOI:** 10.1186/1752-1947-3-114

**Published:** 2009-11-09

**Authors:** Kotb A Metwalley, Hekma S Farghalley, Alaa A Abd-Elsayed

**Affiliations:** 1 Department of Pediatrics, Faculty of Medicine, Assiut University, Assiut, Egypt; 2 Department of Pediatrics, Al-Mabarah Hospital, Assiut, Egypt; 3 Department of Public Health and Community Medicine, Faculty of Medicine, Assiut University, Assiut, Egypt

## Abstract

**Introduction:**

Trisomy 18 is the second most common autosomal trisomy after Down syndrome (trisomy 21). A variety of anomalies of the central nervous system are observed in cases of trisomy 18. The association between trisomy 18 and congenital hydrocephalus is very rare.

**Case presentation:**

A 4-month-old male Egyptian baby boy was referred to Assiut University hospital for evaluation of his large-sized head. The initial clinical examination revealed facial dysmorphism including a prominent wide forehead, wide anterior fontanel, bushy eyebrows, synophrosis, small palpebral fissures, ocular hypertelorism, high arched palate, depressed nasal bridge, low-set ears, micrognathia, bilateral clenched hands with over lapping fingers, rocker-bottom feet and penile hypospadius. A computed tomography scan of the patient's head showed a dilatation of all the ventricular systems of the brain that suggested hydrocephalus. A chromosome analysis of his peripheral blood confirmed a trisomy of chromosome 18 (47, XX+18). The hydrocephalus was treated with a ventriculoperitoneal shunt because of the abnormal increase in his head circumference. He was discharged home on nasogastric feeds at the age of 5 months. Despite the advice of the medical team, his parents did not bring him for further follow up. He died at the age of 7 months due to a sudden cardiorespiratory arrest at home.

**Conclusion:**

Microcephaly is not mandatory for the diagnosis of trisomy 18 syndrome because some cases of trisomy 18 can be associated with other anomalies of the central nervous system, including hydrocephalus. There is no proven explanation for this association, and the management of hydrocephalus in such a situation is not different from the usual course of management.

## Introduction

Trisomy 18 (T18) is a well-known malformation syndrome characterized by severe psychomotor and growth retardation, microcephaly, microphthalmia, malformed ears, micrognathia, skeletal and cardiac defects and urogenital anomalies. It was first described in 1960 by Edwards *et al*. [1], and, therefore, also named as Edward's syndrome. The birth prevalence of this disorder is approximately 1 in 3,000 to 1 in 8,000, and the life span of the majority of patients with this condition is less than 1 year [2]. The majority of T18 individuals acquire the syndrome as a result of non-disjunction and rarely due to translocation. The extra chromosome 18 is of maternal origin in 90% to 97% of reported cases and of paternal origin in 3% to 10% of reported cases [3]. Patients with T18 are generally microcephalic [4]. Other reported anomalies of the central nervous include anencephaly [5], Type II Arnold-Chiari malformation [6], enlarged cisterna magna, choroid plexus cysts [2] and holoprosencephaly [7].

## Case presentation

A 4-month-old Egyptian baby boy was referred to Assiut University hospital by a local primary health care centre for further assessment because of his dysmorphic features and large-sized head. He was delivered at home by a midwife at full term through normal vaginal delivery. He had no prenatal or antenatal care. He was the second-born male child to non-consanguineous Egyptian parents who lived in a very small village. The couple also had a 3-year-old son who was completely healthy. The father and mother were 46 and 42 years old, respectively, at the time of the patient's birth. The mother reported that he was smaller in size at birth in comparison with his sibling. At the time of presentation, he had difficulty feeding, vomited frequently and lacked the ability to control his head. He also seldom smiled at his mother, but he had no history of convulsion or disturbed conscious level.

**Figure 1:**
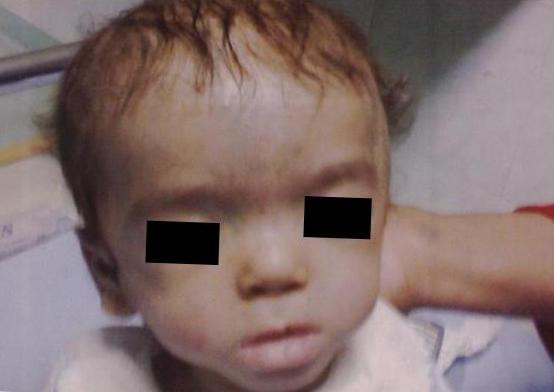
**An image of the patient's bilateral clenched hands**.

**Figure 2:**
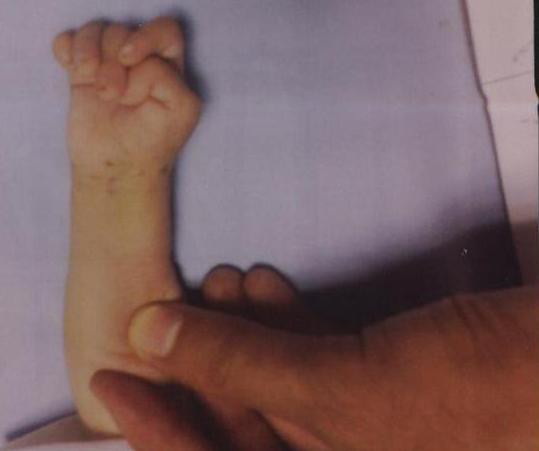
**An image of the patient's overlapping fingers**.

**Figure 3:**
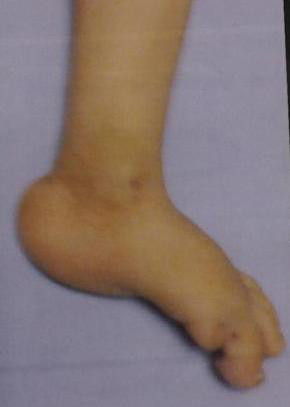
**An image of the patient's rocker-bottom feet**.

**Figure 4:**
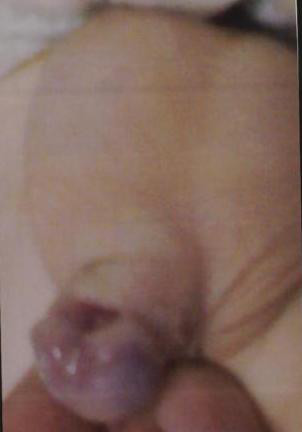
**An image of the patient's penile hypospadius**.

At the initial clinical examination, the patient weighed 4250 g (<5 centile), his head circumference was 47 cm (>97 centile) and his body length was 52 cm (<5 centile). He also had facial dysmorphism including a prominent and wide forehead, wide anterior fontanel, bushy eyebrows, synophrosis, small palpebral fissures, ocular hypertelorism, high arched palate, depressed nasal bridge, low-set ears, micrognathia (Figure 1), bilateral clenched hands with overlapping fingers (Figure 2), rocker-bottom feet (Figure 3), and penile hypospadius (Figure 4).

Cardiac examination revealed that the patient had a Grade III pansystolic murmur over his left lower sternal area with no signs of heart failure. Neurological examination revealed an increased tone in his upper and lower limbs with overactive tendon reflexes. Further tests showed that his hemogram, electrolytes, renal and liver functions, blood glucose, chest X-ray and abdominal ultrasound were all normal. Echocardiography showed that the patient had a small perimembranous ventricular septal defect (VSD). A computed tomography (CT) scan of his head showed dilatation of all the ventricular systems of his brain and suggested hydrocephalus.

A chromosome analysis (karyotyping) of the patient's peripheral blood confirmed that he had a trisomy of chromosome 18 (47, XX+18). The patient's hydrocephalus was treated with a ventriculoperitoneal shunt because of the abnormal enlargement of his head circumference. He was discharged home on nasogastric feeds at the age of 5 months. Despite the advice of the medical team, however, his patients did not bring the child for further follow up. He died at the age of 7 months due to a sudden cardiorespiratory arrest at home.

## Discussion

T18 is manifested by a variety of anatomic abnormalities involving almost all organ systems as well as life-threatening abnormalities. These abnormities may be caused by the abnormal expression of development of important genes on chromosome 18 within 20 to 23 weeks gestation.

The risk of T18 is known to increase as the age of the child's mother at the time of pregnancy increases [8]. The risk of T18 is also associated with increasing paternal age; however, once maternal age is taken into consideration the association with paternal age disappears [9]. This theory is consistent with our case where both parents were middle aged.

The case we have presented exhibited most of the clinical patterns of T18, namely small palpebral fissures, ocular hypertelorism, high arched palate, depressed nasal bridge, low-set ears, micrognathia, bilateral clenched hands with overlapping fingers, rocker-bottom feet, penile hypospadius, small perimembranous VSD, failure to thrive, hypertonia and developmental retardation. Despite the absence of information regarding birth weight because of his delivery at home, the mother reported that the baby was smaller than her other child had been, which can be taken as an indication of possible intrauterine growth retardation. The observed malformations of the patient are consistent with descriptions of T18 in the literature. Our patient did not present with other brain malformations or diseases except congenital hydrocephalus, which is a very unusual presentation of T18. Whether this congenital hydrocephalus is a new variant or just an independent coincidence remains to be determined.

Hydrocephalus leads to an increase in the size of cerebrospinal fluid spaces, which is also associated with an increase in intracranial pressure (ICP) [10]. Although the underlying cause in acquired hydrocephalus is already generally determined, the etiology of congenital hydrocephalus is still not well established [11]. Among 118 cases of T18 cited in one study [4], 20.3% of them had central nervous system (CNS) anomalies (microcephaly, hydrocephaly and trigonocephaly). The exact number of cases of hydrocephaly, however, was not reported and only one of the cases studied survived up to the age of 7 months, which is similar to the age of death of our patient in this case. Survival in T18 is related to the severity of congenital malformations and, to some extent, the availability and accessibility of pediatric care for the patient.

An ultrasound showing abnormal results is the most sensitive screening test for the prenatal diagnosis of T18 [12]. The mother of our patient was not examined by ultrasonography during her pregnancy.

## Conclusion

Microcephaly is not mandatory for the diagnosis of T18 syndrome because some cases of T18 can be associated with other anomalies of the CNS including hydrocephalus, as in this case. There is no proven explanation for this association, and the management of hydrocephalus in such a situation is not different from the usual course of management.

## Abbreviations

CNS: central nervous system; CT: computed tomography; T18: trisomy 18; VSD: ventricular septal defect; ICP: intracranial pressure

## Consent

Written informed consent was obtained from the patient's parents for publication of this case report and any accompanying images. A copy of the written consent is available for review by the Editor-in-Chief of this journal.

## Competing interests

The authors declare that they have no competing interests.

## Authors' contributions

KA and HF diagnosed, investigated, followed up and managed the patient. KA also drafted the manuscript. AE also managed the patient and carried out general coordination for this case report. AE also drafted the manuscript, wrote its final version and provided important suggestions as regards intellectual content. All authors read and approved the final manuscript.
